# In Vitro Antimicrobial Activity of Gel Containing the Herbal Ball Extract against *Propionibacterium acnes*

**DOI:** 10.3390/scipharm86010008

**Published:** 2018-02-28

**Authors:** Chutima Jantarat, Pornpak Sirathanarun, Tatsanee Chuchue, Atthaphon Konpian, Gorawit Sukkua, Prutthicha Wongprasert

**Affiliations:** 1 Drug and Cosmetics Excellence Center, Walailak University, Thasala, Nakhon Si Thammarat 80160, Thailand; 2 School of Pharmacy, Walailak University, Thasala, Nakhon Si Thammarat 80160, Thailand; spornpak@wu.ac.th (P.S.); tatsanee.ch@wu.ac.th (T.C.); 55144646@wu.ac.th (A.K.); Gorawit.su@mail.wu.ac.th (G.S.); 55145551@wu.ac.th (P.W.)

**Keywords:** herbal ball, gel, *Propionibacterium acnes*, antimicrobial activity, *Andrographis paniculata*, *Centella asiatica*, Benchalokawichian remedy, *Hesperethusa crenulata*

## Abstract

The herbal ball has been used as a Thai traditional medicine for relieving many diseases including acne. However, the application process of the herbal ball in practice is complicated and time consuming. The objective of this work was to utilize an herbal ball extract to formulate a gel to reach a more favorable use of the herbal ball for acne treatment. An herbal ball consisting of *Andrographis paniculata*, *Centella asiatica*, the Benchalokawichian remedy and the stem bark powder of *Hesperethusa crenulata* was prepared. The obtained herbal ball was steamed and squeezed to obtain the extract. Gel formulations containing the herbal ball extract at concentrations of 0.1, 1 and 5% *w*/*w* were prepared based on a carbomer gel. The herbal ball extract had antioxidant (EC_50_ = 219.27 ± 36.98 μg/mL) and anti *Propionibacterium acnes* activities (minimum inhibitory concentration (MIC) and minimum bactericidal concentration (MBC) = 31.25 μg/mL). The 5% *w*/*w* gel formulation had antimicrobial activity against *P. acnes*, showing an inhibition zone value of 10.00 ± 1.00 mm. This indicates that the developed gel formulation has potential for acne treatment. In comparison to the traditional method of herbal ball usage, the application of herbal ball extract in the form of gel should be more convenient to use.

## 1. Introduction

Acne vulgaris (acne) is one of the most common skin disorders in the general population around the world. In Thailand, it has been found to be the highest incidence of out-patients seeing dermatologists from 2013–2015 according to a study by the Institute of Dermatology, Thailand [1]. Acne affects more than 80% of adolescents and young adults and can also be seen in individuals in both adults and children [2]. Although it is not life threatening, acne often causes physical and psychological problems such as poor self-image, depression and anxiety. Acne is caused by many factors related to the hyperactivity of the sebaceous gland, follicular epidermal hyperproliferation and inflammation caused by pathogens. The well-known pathogen linked to the skin condition of acne is *Propionibacterium acnes* (*P. acnes*), an anaerobic, gram-positive bacterium. 

There are a variety of effective acne treatments. Topical therapy is considered as the standard treatment for mild and moderate acne. Some common medications include benzoyl peroxide, antibiotics, retinoids and salicylic acid. Oral antibiotics are suitable for the treatment of moderate and severe acne. Some antibiotic therapies for acne include tetracycline, doxycycline, minocycline, erythromycin, trimethoprim-sulfamethoxazole, trimethoprim and azithromycin [1]. However, the use of antibiotics for acne treatment causes bacterial resistance. The prevalence of *P. acnes* resistant to clindamycin, tetracycline, doxycycline and erythromycin has occurred in many countries worldwide [3]. Isotretinoin, a group of retinoids, is an efficacious treatment for severe acne; however, the application of this treatment when pregnant must be avoided as it can cause abortion and birth defects. Phytotherapy is an alternative treatment for acne and has gained more attention recently due to several advantages such as low side effects, its availability in local areas and is normally low cost. Many herbs in Thailand have been proven to be able to treat acne—for example, *Centella asiatica*, *Zingiber montanum*, *Curcuma longa*, *Garcinia mangostana* and *Andrographis paniculata* [4,5]. These have activity in different mechanisms including antibacterial, antioxidant, and/or anti-inflammation effects [6]. Benchalokawichian, a Thai herbal remedy, is one of the medicines listed in the Thailand National List of Essential Medicines that is typically used for relief of fever [7]. It is composed of roots from five plants including *Ficus racemosa*, *Capparis micracantha*, *Clerodendrum petasites*, *Harrisonia perforate* and *Tiliacora triandra* in equal amounts of each. Recently, it has also been used to treat acne and several skin disorders [8]. Several herbs may be used in combination to obtain better result and has nowadays been developed for use in the form of an herbal ball.

**Figure 1:**
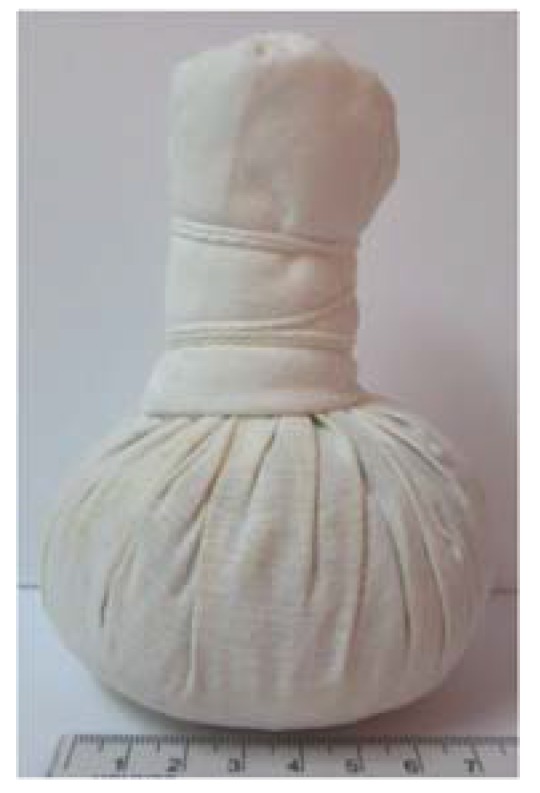
Herbal ball.

The herbal ball or herbal compress ball (Figure 1) is a Thai traditional treatment that uses a round herbal ball of selected herbs. The selected herbs can be used either in fresh or dried forms. The herbal ball has been used for relieving many diseases including acne. Its pharmacological activity depends on the herb composition. To prepare an herbal ball, selected herbs are chopped into small pieces and wrapped in a muslin cloth and bundled together as a sphere. To use the herbal ball, it must be steamed for about 15–20 min, then applied onto the skin with a gentle pressing action to allow the active ingredients to be absorbed [9]. The herbal ball treatment is very popular in spas, especially in the case of inducing deep relaxation, relieving stress and fatigue. However, for treating chronic diseases including acne, the herbal ball treatment must be applied every day for a long time, so it is not suitable as it takes many processes and time for treatment. One herbal ball can be used repeatedly only 3–4 times before being discarded. After the first use, it must be stored in a refrigerator until the next time of use. Therefore, the adaptation of using an herbal ball from the traditional method to a formulation containing the herbal ball extract would be more convenient.

The objective of this work was to prepare a gel formulation containing the herbal ball extract to be used instead of using the herbal ball in the traditional method for acne treatment. The herbal ball contained four types of herbs including *Andrographis paniculata*, *Centella asiatica*, the Benchalokawichian remedy and the stem bark powder of *Hesperethusa crenulata* (Thanaka), which have different mechanisms to treat acne. *Andrographis paniculata* and *Centella asiatica* have antibacterial and antioxidant effects [6], the Benchalokawichian remedy has an antibacterial effect [10] and Thanaka has anti-inflammatory and antioxidant effects [11]. The herbal ball was extracted in the same way as the traditional use. The obtained extract was further used to prepare the gel formulation and the gel formulation was tested for physicochemical properties and anti *P. acnes* activity.

## 2. Materials and Methods

### 2.1. Materials

2,2-Diphenyl-1-picrylhydrazyl (DPPH), gallic acid and tetracycline were purchased from Sigma-Aldrich (St. Louis, MO, USA). Carbopol^®^ 940 was supplied by BF Goodrich (Cleveland, OH, USA). Propylene glycol and triethanolamine were supplied from Fisher Scientific (Leicestershire, UK). *Propionibacterium acnes* (DMST 14916) was obtained from the Department of Medical Science, Ministry of Public Health, Thailand. Brain heart infusion (BHI) media were purchased from Himedia laboratories (Mumbai, India). Clindalin^®^ gel (clindamycin 1% gel, Union Drug Laboratories Ltd., Bangkok, Thailand) was purchased from a drugstore. Distilled and sterile water was used across the study. All other chemicals and solvents were of analytical grade and used as received.

*Andrographis paniculata* and *Centella asiatica* leaves were collected as fresh herbs from the Krasaesin district, Songkhla, Thailand. The stem bark powder of *Hesperethusa crenulata* or Thanaka was obtained from the Thongphaphum district, Kanchanaburi, Thailand. The Benchalokawichian remedy was purchased as the dried powder from the Thaprachan herb shop, Bangkok, Thailand. 

### 2.2. Preparation of Herbal Ball and Herbal Ball Extract

The herbal ball was prepared by using four types of dried herbs including *Andrographis paniculata* leaves, *Centella asiatica* leaves, the Benchalokawichian remedy in powder form and Thanaka. Fresh leaves of *Andrographis paniculata* and *Centella asiatica* were cleaned and dried in a hot air oven (UF 110, Memmert GmbH + Co. KG, Schwabach, Germany) at 45 °C, then roughly ground with a mortar and pestle. Dried herbs (10 g each) were mixed together and wrapped in a muslin cloth and tied up together in a spherical shape. The herbal ball was then moistened by dipping into water for 2–3 s and steamed by placing over hot water in a water bath (WNB 29, Memmert GmbH + Co. KG) at 80 °C for 15 min. Subsequently, the herbal ball was squeezed by hand to obtain the crude extract solution. The herbal ball was repeatedly extracted another three times. The obtained extract solution was poured together and centrifuged at 9000 rpm at 25 °C for 5 min. The supernatant was filtered through Whatman^®^ grade No. 1 filter paper and dried by freeze-dryer (GAMMA 2-16 LSCplus, Martin Christ Gefriertrocknungsanlagen GmbH, Osterode, Germany). After freeze-drying, a dried herbal ball extract of 6.74% of the total weight of the dried herbs was obtained. The dried herbal ball extract was kept in the desiccator until used.

### 2.3. Evaluation of Herbal Ball Extract

#### 2.3.1. Antioxidant Activity

The antioxidant activity of the herbal ball extract was evaluated by the DPPH assay. The procedures were conducted in the same manner as described by Sharma et al. [12]. The DPPH was dissolved in ethanol and further diluted to make the final concentration of 0.4 mM. The sample solution was prepared by dissolving the herbal ball extract in water and diluted to obtain five concentrations from 10 to 300 μg/mL. Gallic acid was used as the positive control. It was dissolved in ethanol and diluted to obtain five concentrations from 1 to 8 μg/mL. A 100 μL aliquot of each sample solution was added to 100 μL of DPPH solution in a 96-well plate. The solution was mixed by being gently shaken and kept in the dark for 30 min. The absorbance at 517 nm was measured using a microplate reader (BioTek, BioTek Instrument, Inc., Winooski, VT, USA). The antioxidant activity percentage (AA%) was evaluated using the following equation:

(1) $$\mathrm{AA}\%=(1-A_{s}/A_{0})\times 100$$

where $A_{s}$ and $A_{0}$ are the absorbance at 517 nm of sample solution and blank DPPH solution, respectively.

The half maximal effective concentration (EC_50_), which is the concentration of the sample exhibiting 50% antioxidant activity, was calculated from the curve plotting between the sample concentration (*x* axis) and the AA% (*y* axis) [13].

#### 2.3.2. Anti *Propionibacterium acnes* Activity

The anti *P. acnes* activity of the herbal ball extract was tested by determining the minimum inhibitory concentration (MIC) by the broth dilution method and the minimum bactericidal concentration (MBC) following the MIC assay. *P. acnes* were grown in a BHI broth under anaerobic conditions using the Anaerocult A Mini system (Merck, Darmstadt, Germany) at 37 °C for 72 h. The turbidity of bacterial suspension was adjusted by using McFarland No. 0.5 to obtain about 1 × 10^8^ colony-forming unit (CFU)/mL. The herbal ball extract (0.05 g) was dissolved in sterile water and the volume adjusted to 100 mL to obtain a concentration of 500 μg/mL as a stock solution. The stock solution was filtered through a 0.45 μm sterile filter before being diluted with BHI broth in a 2-fold dilution series in glass test tubes at six concentrations from 250 to 7.81 μg/mL. The positive control was tetracycline, which was diluted in a 2-fold dilution series to achieve six concentrations from 0.24 to 0.0075 μg/mL. The *P. acnes* suspension (50 μL) was added in each sample solution (1 mL). The samples were then incubated in an anaerobic environment using the Anaerocult A Mini system (Merck, Darmstadt, Germany) at 37 °C for 48 h. The lowest concentration with a clear culture observed was detected as the MIC. Negative controls were performed with the same procedure as the sample but without adding the *P. acnes*. 

For determination of MBC, the dilution representing the MIC and three of the more concentrated sample dilutions were plated on a BHI agar. A sample dilution of one of the less concentrated MIC sample dilutions was used as the positive control. The agar plates were incubated in an anaerobic environment using the Anaerocult A Mini system (Merck, Darmstadt, Germany) at 37 °C for 48 h. The lowest concentration of the sample that showed no visible growth was detected as the MBC.

### 2.4. Preparation of Gel Formulation Containing the Herbal Ball Extract

Gel formulations containing the herbal ball extract were prepared in three concentrations including 0.1, 1 and 5% *w*/*w* using carbomer as the gel base. The herbal ball extract (0.1, 1, or 5 g) was dissolved in about one-third of the sterile water used in the formula. The herbal ball extract solution was filtered through a 0.45 μm sterile filter and mixed with another part of sterile water to obtain a solution with a total volume of 95 mL. Carbopol^®^ 940 (1 g) and propylene glycol (3 mL) were mixed in a mortar. The herbal ball extract solution was then added and stirred continuously to ensure complete hydration. Triethanolamine (about 1–2 mL) was then added dropwise under gentle stirring until a gel was formed.

### 2.5. Evaluation of Gel Formulation Containing the Herbal Ball Extract

#### 2.5.1. Physicochemical Properties

Gel formulations containing the herbal ball extract were subjected to tests for appearance, color, odor, homogeneity and pH. The appearance, color and homogeneity were tested by visual inspection. The homogeneity was graded as +++ good, ++ fair and + poor. The pH was determined using a digital pH meter (Cole-Parmer, London, UK).

#### 2.5.2. Anti *Propionibacterium acnes* Activity

The anti *P. acnes* activity of the gel formulations containing the herbal ball extract was evaluated by using the agar well diffusion method. The BHI agar was inoculated by spreading *P. acnes* over the entire agar surface and left to dry. A hole with a diameter of 6 mm was punched aseptically with a sterile tip. The gel formulations were introduced into the well. Then, agar plates were incubated in an anaerobic environment using the Anaerocult A Mini system (Merck, Darmstadt, Germany) at 37 °C for 48 h. Gel base and Clindalin^®^ gel (clindamycin 1% gel) were used as the placebo and positive control, respectively. The anti *P. acnes* activity was investigated as the diameters of the clear zone.

#### 2.5.3. Stability Study

The gel formulations were subjected to accelerated stability tests by using a freeze-thaw cycle for six cycles. For each cycle, the samples that were packed in the plastic tube were stored at 4 °C for 24 h, then stored at 30 °C for 24 h. After the completion of six cycles, the gel formulations were evaluated for physicochemical properties when compared to the freshly prepared formulations.

## 3. Results and Discussion

### 3.1. Antioxidant Activity of the Herbal Ball Extract

**Figure 2:**
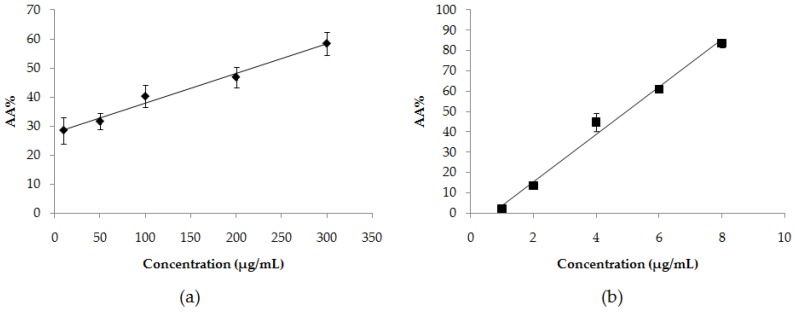
Antioxidant activity percentage (AA%) of (**a**) the herbal ball extract and (**b**) the positive control gallic acid at various concentrations.

The DPPH assay is a reliable method to determine the antioxidant activity of the samples. The antioxidants can stabilize the free DPPH radicals due to the proton donating ability. The reduction of free DPPH radicals can be detected by spectroscopy at 517 nm based on the purple color changing to yellow. In this study, the herbal ball extract was sterilized by being filtered through a 0.45 μm sterile filter before testing. By ignoring sterilization, microbial contaminants of the sample might be obtained. The antioxidant activity of the herbal ball extract and the positive control gallic acid are shown in Figure 2. The AA% of the herbal ball extract and gallic acid increased with increasing concentration. The values within the examined concentrations ranged from 25–60% for the herbal ball extract and 2–85% for the gallic acid. In addition, it was observed that the antioxidant activities of both the herbal ball extract and gallic acid within the examined concentrations were related to concentration as a linear relationship. The calculated EC_50_ of the herbal ball extract and gallic acid were 219.27 ± 36.98 μg/mL and 4.97 ± 0.16 μg/mL, respectively. The herbal ball extract had lower antioxidant activity than the gallic acid by about 40-fold. This would be generally observed for the crude plant extract when compared to the pure antioxidant compound. However, when compared to the antioxidant activity of the herbal ball extract obtained from several herbs composition with that of the individual herb extracts reported in the literature, the herbal ball extract had better activity. The EC_50_ value of the DPPH radical scavenging activity of the water extract of *Andrographis paniculata* and *Centella asiatica* were 418.51 μg/mL [14] and 300 μg/mL [15], respectively. This implied that the extract obtained from the herb combination could induce more effective activity than the extract obtained from the individuals. Moreover, Thanaka might also increase the antioxidant activity of the herbal ball extract. Antioxidant activity is one of the mechanisms underlying phytotherapy’s treatment of acne. Although the antioxidant activity of the herbal ball extract was not high, it might help to improve the overall results of acne treatment by synergizing it with anti *P. acnes* activity.

### 3.2. Propionibacterium acnes Activity of Herbal Ball Extract

*P. acnes* is responsible for the inflammation process in acne formation. The herbal ball extract showed anti *P. acnes* activity. The MIC against *P. acnes* was equal to the MBC. The value was 31.25 μg/mL. In comparison to the positive control tetracycline (MIC = 0.06 μg/mL), the activity of the herbal ball extract was about 500-fold lower. It has been recommended that the threshold MIC values for plant extract and pure compounds should be 100 μg/mL and 10 μg/mL, respectively [16]. The herbal ball extract, therefore, could be considered to have high antimicrobial activity against *P. acnes*. When the activity of the herbal ball extract found in this study was compared with its herb components (*Andrographis paniculata* and *Centella asiatica*) reported in the literature, it was found that the herbal ball extract had better activity. The MIC of the water extract of *Andrographis paniculata* and *Centella asiatica* against *P. acnes* were 0.625 mg/mL and 5 mg/mL, respectively [4]. The combination of natural substances with similar biological activity that could provide additive activity against acne-causing bacteria was also found in the study by Julianti et al. [17]. The Benchalokawichian remedy was reported to be effective against a variety of bacteria including both gram positive and negative bacteria [10], however, for antimicrobial activity against *P. acnes*, it has not been reported. The Benchalokawichian remedy might also be beneficial for acne treatment caused by bacteria. The main anti *P. acnes* activity of the herbal ball extract should come from *Andrographis paniculata*, which contains andrographolide, an active principle of *Andrographis paniculata* with antimicrobial activity [18,19]. *Centella asiatica* and the Benchalokawichian remedy would synergize its activity. The activity of the extract from the herbal ball was therefore much greater than the activity from its herbal components. The same amount of MIC and MBC obtained from the herbal ball extract against *P. acnes* suggested that the herbal ball extract could possibly act as a bactericidal agent for this microorganism.

### 3.3. Physicochemical Properties of Gel Formulation Containing the Herbal Ball Extract and Stability

Carbomer gel formulations containing the herbal ball extract in three concentrations were successfully prepared. The lowest (0.1% *w*/*w*) and highest (5% *w*/*w*) concentrations were about 32-fold and 1600-fold higher in concentration than the MIC, respectively. The gel formulations were clear and translucent with pale yellow, yellow-brown and dark brown colors for the 0.1, 1 and 5% *w*/*w* formulations, respectively (Table 1). The 5% *w*/*w* formulation had a mild odor while the others were odorless. A visual observation revealed that all formulations were homogeneous. The pH of the formulations was between 5.5 and 7.2. This was due to the neutralization of the carbomer (acid) and triethanolamine (base) and the gel was formed at a pH of around 6 [20]. A pH value of 5.5 in formulations for skin use has been recommended [21]. Therefore, the prepared gel was suitable for skin use.

After a freeze-thaw cycle for six cycles, the physicochemical properties of the gel formulations in term of appearance, color, odor, homogeneity and pH were unchanged. Therefore, the prepared gel formulations were physically stable in accelerated conditions.

### 3.4. Anti Propionibacterium acnes Activity of Gel Formulation Containing the Herbal Ball Extract

**Figure 3:**
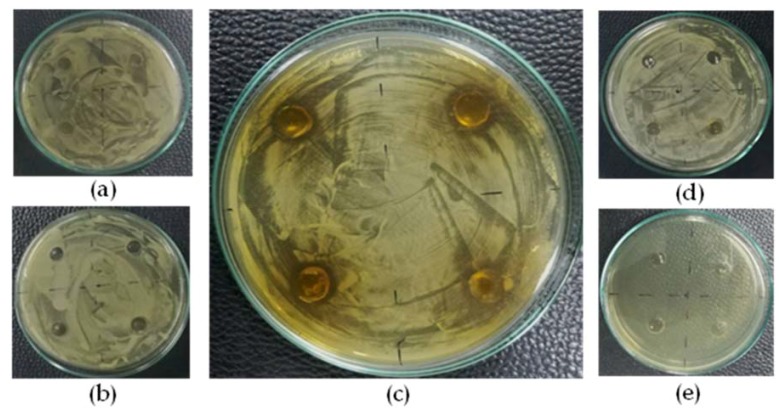
Photographs showing the inhibition zone of gel formulation containing the herbal ball extract at (**a**) 0.1% *w*/*w*, (**b**) 1% *w*/*w* and (**c**) 5% *w*/*w* against *P. acnes* when compared with that of (**d**) the placebo and (**e**) the positive control.

The gel formulation containing the herbal ball extract at a concentration of 5% *w*/*w* only exhibited antimicrobial activity against *P. acnes* with a clear zone diameter 10.00 ± 1.00 mm (Figure 3), while the lower concentration gel formulations (0.1 and 1% *w*/*w*) could not inhibit the growth of *P. acnes*. The placebo had no activity to inhibit the growth of *P. acnes* while the Clindalin^®^ gel (positive control) could effectively inhibit the growth of *P. acnes* as no *P. acnes* was observed on the whole surface of the agar plate. The anti *P. acnes* activity of the gel formulations was relatively low although the herbal ball extract at high concentrations of about 1000-fold of MIC was used. This might be due to the effect of the gel base, which retarded the diffusion of the herbal ball extract into the agar. The agar well diffusion method could be used for only screening the antimicrobial activity of the gel formulation. In order to effectively use this formulation for acne treatment in practice, the improvement of its activity and testing more *P. acnes* strains should be further investigated.

## 4. Conclusions

Gel formulations containing the herbal ball extract with various amounts of the herbal ball extract were prepared in an attempt for use instead of the traditional method of the herbal ball for acne treatment. The gel formulations were subjected for antimicrobial activity against *P. acnes*. The herbal composition of the herbal ball included *Andrographis paniculata*, *Centella asiatica*, the Benchalokawichian remedy and the stem bark power of *Hesperethusa crenulata* (Thanaka), which have different mechanisms for treating acne. The herbal ball extract had good activities for antioxidants and anti *P. acnes* according to the DPPH assay and broth dilution study. The gel formulation containing the herbal ball extract with the concentration of 5% *w*/*w* showed antimicrobial activity against *P. acnes* according to the agar well diffusion study. The obtained results suggest that gel formulation containing an herbal ball extract of at least 5% *w*/*w* could be effectively used for acne treatment. Importantly, the prepared gel was more convenient to use in comparison to the traditional method of herbal ball usage.

## Figures and Tables

**Table 1:** Physicochemical properties of gel formulations containing the herbal ball extract at the concentration of 0.1, 1 and 5% *w*/*w*.

Properties		Formulations	
	0.1% *w*/*w*	1% *w*/*w*	5% *w*/*w*
Appearance	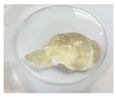	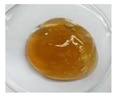	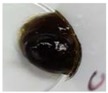
Color	Pale yellow	Yellow-brown	Dark brown
Odor	-	-	Mild odor
Homogeneity	+++	+++	+++
pH	5.58 ± 0.02	6.61 ± 0.02	7.24 ± 0.01

Note: homogeneity was graded as +++ good, ++ fair and + poor.
